# Distinct Patterns of Association of Variants at 11q23.3 Chromosomal Region with Coronary Artery Disease and Dyslipidemia in the Population of Andhra Pradesh, India

**DOI:** 10.1371/journal.pone.0153720

**Published:** 2016-06-03

**Authors:** Rayabarapu Pranav Chand, Arramraju Sreenivas Kumar, Kapadia Anuj, Satti Vishnupriya, Battini Mohan Reddy

**Affiliations:** 1 Molecular Anthropology Group, Indian Statistical Institute, Hyderabad, India; 2 Department of Cardiology, Care Hospitals, Hyderabad, India; 3 Department of Genetics, Osmania University, Hyderabad, India; Sudbury Regional Hospital, CANADA

## Abstract

In our attempt to comprehensively understand the nature of association of variants at 11q23.3 apolipoprotein gene cluster region, we genotyped a prioritized set of 96 informative SNPs using Fluidigm customized SNP genotyping platform in a sample of 508 coronary artery disease (CAD) cases and 516 controls. We found 12 SNPs as significantly associated with CAD at P <0.05, albeit only four (rs2849165, rs17440396, rs6589566 and rs633389) of these remained significant after Benjamin Hochberg correction. Of the four, while rs6589566 confers risk to CAD, the other three SNPs reduce risk for the disease. Interaction of variants that belong to regulatory genes *BUD13* and *ZPR1* with *APOA5*-*APOA4* intergenic variants is also observed to significantly increase the risk towards CAD. Further, ROC analysis of the risk scores of the 12 significant SNPs suggests that our study has substantial power to confer these genetic variants as predictors of risk for CAD, as illustrated by AUC (0.763; 95% CI: 0.729–0.798, p = <0.0001). On the other hand, the protective SNPs of CAD are associated with elevated Low Density Lipoprotein Cholesterol and Total Cholesterol levels, hence with dyslipidemia, in our sample of controls, which may suggest distinct effects of the variants at 11q23.3 chromosomal region towards CAD and dyslipidemia. It may be necessary to replicate these findings in the independent and ethnically heterogeneous Indian samples in order to establish this as an Indian pattern. However, only functional analysis of the significant variants identified in our study can provide more precise understanding of the mechanisms involved in the contrasting nature of their effects in manifesting dyslipidemia and CAD.

## Introduction

Coronary artery disease (CAD) is most predominant and ranks as number one in causing deaths due to cardiovascular diseases (CVDs) in India. Dyslipidemia, diabetes, hypertension, smoking and obesity or overweight were identified as traditional risk factors of CAD across the ethnic groups [1]. The disease occurs due to the process of atherosclerosis, a progressive damage in the blood vessels supplying blood to the heart muscles. The primary event of atherosclerosis is the endothelial injury or dysfunction, which is triggered by abnormal lipoprotein metabolism with subsequent dyslipidemia [2]. It is apparent from the candidate gene association studies that relatively greater number of lipoprotein metabolism related genes is observed to be more consistently associated with CAD as compared to the candidate genes related to other metabolisms [3]. Among the genes that regulate this metabolism, apolipoprotein genes that code for cofactors of several enzymes of cholesterol transport system are the key regulators. These genes are primarily found as clusters on chromosome 11 and 19. The candidate gene [4–7] and GWAS studies [8,9] revealed 11q23.3 Apolipoprotein gene cluster region, in particular, to be more often associated with lipid traits. Exclusive attempts were also made to comprehensively understand the role of genetic variants in this lipid influencing region among the Caucasians [10] and north Indian Punjabis [8] and a couple of polymorphisms were found to be associated with high density lipoprotein cholesterol (HDL-C) and plasma triglyceride (TG) concentrations.

Indians are known to have a unique pattern of dyslipidemia, usually characterized by low levels of low density lipoprotein cholesterol (LDL-C), elevated triglycerides and low HDL with predominantly atherogenic and small-dense LDLs [11, 12]. This characteristic feature is referred to as ‘*atherogenic dyslipidemia’* [13, 14]. Although a few of the conventional polymorphisms within the *APOAI-CIII-AIV-AV* gene cluster region were found to be associated with lipid traits [15, 16], a large number of SNPs in this region remained unexplored for their association with CAD as well as with the lipid traits among Indians. On the other hand, with its reported incidence of 67.6% among the CAD cases, dyslipidemia appears to be the primary cause of CAD in southern Indians [17]. Given the characteristic dyslipidemic feature of Indian populations in general and particularly the South Indians, it is imperative to explore the possible association of polymorphisms at 11q23.3 apolipoprotein gene cluster region with CAD among them. Spanning ~200KB, this chromosomal region contains three regulatory protein coding genes—*BUD13*, *ZPR1* and *SIK3*—and four apolipoprotein coding genes—*APOA1*, *APOC3*, *APOA4* and *APOA5*. In the present study, we made an attempt to explore the association pattern of 96 prioritized SNPs of the 11q23.3 region with CAD in the population of Hyderabad, India.

## Materials and Methods

### Ethics statement

The study protocol was approved by the Indian Statistical Institute Review Committee for Protection of Research Risks to Humans. Written informed consent for all the participants is obtained as per the guidelines.

### The study design and population

A total of 1024 individuals including 508 CAD cases and 516 controls, broadly representing the population of Andhra Pradesh, participated in our case-control study. Patients with characteristic symptoms of stable/unstable angina pectoris along with variable degrees (generally >40%) of stenosis in at least one of the major coronary arteries as determined through angiogram were included in the study. Cases with monogenic diseases, valvular heart disease, cardiomyopathy, renal disease, acute and chronic viral or bacterial infections, asthama, tumours or connective tissue diseases and other vascular diseases were excluded from the study. All the cases were evaluated by interventional cardiologists at the CARE Hospitals, Hyderabad, for the above mentioned criteria. Control samples were recruited by conducting free health camps in and around Hyderabad and broadly representing subjects aged above 45 years and with similar ethnic backgrounds as that of the cases. The individuals with characteristic features of any of the above mentioned disease conditions were not included as part of the controls. However, certain proportions of both the case and control subjects were found with T2DM, dyslipidemia and hypertension.

The population of Hyderabad is a conglomeration of people from different parts of the undivided state of Andhra Pradesh and the mother tongue of most of its population is Telugu, one of the four Dravidian languages. It would be also pertinent to note that despite the subdivision of Telugu population into a number of traditionally endogamous castes and sub castes, Reddy et al. [18] observed genetic differentiation among the populations of Andhra Pradesh to be very low and insignificant; the Markov chain Monte Carlo analysis of population structure, which implements model based clustering method for grouping individuals into populations [19, 20], did not reveal any unique population clusters, suggesting high degree of genetic homogeneity.

### Data and sample collection

Data pertaining to present age, sex, age at diagnosis for cases, height, weight, waist circumference, hip circumference and other background information such as history and current status of smoking, alcoholism and food habits were obtained through a detailed questionnaire. The data pertaining to the current status of the subjects on diabetes, dyslipidemia and hypertension were drawn from hospital records for the cases and through personal interviews for controls. About 5-6ml of fasting blood sample was collected peripherally by certified medical lab technicians. Clinical investigations were done for lipid profile and blood sugar for all the samples at Tapadia diagnostic centre, Hyderabad, using AutoAnalyzer.

### DNA Isolation, SNP selection and Genotyping

DNAs were isolated from all the blood samples using phenol chloroform method [21] and quantified with the help of Thermo Scientific Varioskan™ Flash Multimode Reader using Quant-iT™ PicoGreen® dsDNA Assay Kit.

Apolipoproteins act as cofactors and inhibitors of several enzymes of lipoprotein metabolism and also regulate the cholesterol transport system by being components of HDL cholesterol, LDL cholesterol and very low density lipoproteins (VLDL). Mutations in the genes coding for these proteins lead to abnormal lipoprotein metabolism resulting in increased plasma lipid levels which inturn cause dyslipidemia and/or CAD. In addition to the SNPs at *APOAI-CIII-AIV-AV* genes clustered at 11q23.3 chromosomal region, SNPs located at BUD13 and ZPR1 genes were also identified through GWAS [22] as associated with abnormal lipid traits, which have been replicated among Europeans, Chinese and Asian Indians. Except for a couple of conventional polymorphisms related to *APOAI-CIII-AIV-AV* genes there have been hitherto no genetic studies on the significant GWAS identified SNPs among the south Indian populations. Therefore, to comprehensively genotype the variants at 11q23.3 chromosomal region, we gathered information on SNPs pertaining to this region from earlier candidate gene and sequencing studies and from databases particularly EBI-NHGRI GWAS database, HAPMAP and dbSNP. Given the key role of *BUD13* in splicing mechanism and *ZPR1* as essential protein for normal cell proliferation and signal transduction, we also included SNPs related to these regulatory protein coding genes. A total of 130 SNPs, mostly studied through candidate gene and GWAS approaches, were subjected to Fluidigm D3 Assay design software [23] and a panel of 96 SNPs with high efficiency for genotyping was chosen (S1 Table). Most of these SNPs were characterized as intronic/intergenic and none were studied for their functional role in the manifestation of dyslipidemia or coronary artery disease, except for rs964184 which is a 500BP downstream variant of ZPR1 gene.

Genotyping was performed using fluidigm nanofluidic SNP genotyping system. Eleven 96.96 IFC chips were utilized for genotyping wherein the selected 96 SNPs were analyzed against 96 samples in each chip. These chips were thermal cycled and the end-point fluorescent values were measured on Biomark^TM^ system. Final sample wise genotype calls were obtained using Fluidigm SNP Genotyping Analysis software. A subset of 240 samples was genotyped prior to genotyping of the total 1024 samples. The observed call concordance was 100%. Prior to genetic association analysis, data quality control was achieved by limiting the sample wise call rate to ≥90%. This resulted in genotype call rate of 99% for 386 cases and 462 controls, which are considered for further analysis. Further, of the 96 SNPs analyzed, only 75 SNPs were qualified for final analysis, after excluding SNPs that either showed minor allele frequency < 1% or deviated from hardy Weinberg equilibrium (P<0.001). However, we furnished the allele and genotypic data for the entire set of SNPs and for all the cases and controls in S2 Table.

### Statistical Methods

The descriptive statistical analysis of the background data on quantitative variables was done using MINITAB (version 17). The significance of the mean difference between two groups was obtained using students T-test. Genotyping quality check and association analysis of alleles were done using PLINK [24]. Genotype-phenotype association analysis assuming different genetic models-dominant, co-dominant, recessive, over dominant and log-additive- and logistic regression analysis with covariates were performed using ‘SNPassoc’ package of R PROGRAM. Analysis of the cumulative effect of risk alleles was done with the help of Microsoft EXCEL. The power of our study in discriminating CAD (Receiver Operating Characteristic curve) based on the cumulative risk scores was estimated using SPSS (Version 21, IBM SPSS). Linkage disequilibrium and haplotype association analyses were done using HAPLOVIEW (Version 4.2). RevMan version 5.3 was used for Meta-analysis.

## Results

The means and SDs of age, age at onset of the disease, body mass index (BMI), waist hip circumference ratio (WHR), systolic blood pressure (SBP), diastolic blood pressure (DBP), fasting blood sugar (FBS) and lipid profile are presented separately for CAD patients and controls in Table 1. While the mean values of BMI, DBP, TC, HDLC, LDLC are significantly lower (P = 0.015, 0.028, 0.0001, 0.0001, 0.0001, respectively), the mean systolic blood pressure (P<0.007) and fasting blood sugar (P<0.0001) are significantly higher among the CAD patients. The elevated triglyceride levels with optimum TC and LDLC of the controls represent characteristic atherogenic dyslipidemic feature of the general south Indian population. However, we found average LDLC and HDLC levels to be lower than the NCEP ATPIII defined normal values for the CAD patients. This trend is similar to that observed in a study of large cohort of US CAD subjects, which analyzed admission levels of LDLC and HDLC while adjusting for confounding factors, particularly the lipid lowering medication history before admission [25]. Unfortunately, we could not obtain data on medication, which would have enabled us to explain our findings more specifically.

**Table 1:** Baseline characteristics of the case and control samples of the present study.

Variable	Mean ± SD		P Value(T-Test)
	CASES(n = 386)	CONTROLS(n = 462)	
Age	55.4 **±** 10.1	50.74 **±** 9.8	<0.0001^*^
Height	161.8 **±** 7.6	158.6 **±** 9.2	<0.0001^*^
Weight	68.1 **±** 10.7	67.6 **±** 13.5	0.65
BMI	26.02 **±** 3.8	26.9 **±** 4.5	0.015^*^
Waist Circumference	NA	91.2 **±** 12.2	NA
Hip Circumference	NA	97.5 **±** 12.1	NA
WHR	NA	0.94 **±** 0.1	NA
SBP	131.1 **±** 16.7	127.3 **±** 14.5	<0.007^*^
DBP	81.5 **±** 10.3	83.4 **±** 9.2	<0.029^*^
FBS(mg/dl)	150.9 **±** 62.4	96.5 **±** 41.0	<0.0001^*^
TC(mg/dl)	153.6 **±** 33.6	190.6 **±** 38.7	<0.0001^*^
TG(mg/dl)	148.4 **±** 87.9	161**±** 107	0.06
HDLC(mg/dl)	41.3 **±** 2.2	47.7 **±** 30.5	<0.0001^*^
LDLC(mg/dl)	84.7 **±** 28.1	113.6 **±** 33.3	<0.0001^*^
VLDL(mg/dl)	29.7 **±** 18.9	32.1 **±** 21.4	0.086

BMI–Body Mass Index, SBP–Systolic Blood Pressure, DBP–Diastolic Blood Pressure, FBS–Fasting Blood Sugar, TC–Total Cholesterol, TG–Triglycerides, HDLC–High Density Lipoprotein Cholesterol, LDLC–Low Density Lipoprotein Cholesterol, VLDL–Very Low Density Lipoprotein, WHR–Waist to Hip Ratio

* indicates significant P values, NA–Not available.

### The allelic association with CAD

The association analysis of 75 SNPs which are in HWE suggests 12 SNPs as significantly associated with CAD at P <0.05 albeit only four of these remained significant after Benjamin Hochberg correction for multiple correction. The nearby genes to which the associated SNPs belong, allelic frequencies, chi-square values, and odds ratios are presented in Table 2. The odds ratios from the logistic regression analysis suggest that the minor alleles of the following SNPs- rs1263171-**A**, rs664059-**T**, rs10488699-**A**, rs6589566-**G** and rs2075294-**T**are associated with increased risk, whereas rs5072-**T**, rs2849165-**A**, rs633389-**T**, rs1263163-**A**, rs7396835-**T**, rs17440396-**A** and rs2187126-**G**are associated with decreased risk for CAD. While five of the above 12 SNPs (rs2849165, rs633389, rs1263163, rs1263171, rs7396835) belong to intergenic region of *APOA5*-*APOA4* genes, one SNP (rs5072) belongs to intronic region of *APOA1* gene. Of the remaining, four SNPs (rs17440396, rs664059, rs10488699, rs2187126) belong to intronic regions of *BUD13* and two (rs6589566 and rs2075294) belong to *ZPR1* regulatory gene. However, out of the most prominent four SNPs (rs2849165, rs17440396, rs6589566 and rs633389) that remained significant even after Benjamin Hochberg correction, only one (rs6589566) appears to confer significant risk to CAD, while the others seem to reduce risk for the disease.

**Table 2:** Allelic association of variants at 11q23.3 chromosomal region with CAD along with chi-square values and odds ratios from logistic regression, before and after adjusting for covariates- age, sex and quantitative lipid traits.

SNP Rs ID	Gene/Function of SNP	Alleles	Minor Allele Frequency		Χ^2^	Unadjusted		Adjusted for Age, Sex and Lipid traits	
		Minor/Major	CASES	CONTROLS		P-Value	OR (CI 95%)	P-Value	OR (CI 95%)
rs5072	APOA1/ Intronic	T/C	0.32	0.37	4.23	0.04	0.8	0.02	0.7
							(0.65–0.99)		(0.56–0.96)
rs2849165*	APOA5-APOA4/ Intergenic	A/G	0.22	0.36	40.05	2.48x10^-10^	0.49	1.2x10^-8^	0.41
							(0.39–0.61)		(0.31–0.56)
rs633389*	APOA5-APOA4/ Intergenic	T/C	0.08	0.16	21.9	2.80X10^-06^	0.47	0.13^#^	0.73
							(0.35–0.65)		(0.49–1.09)
rs1263163	APOA5-APOA4/ Intergenic	A/G	0.15	0.21	8.15	0.004	0.69	0.88^#^	0.97
							(0.53–0.89)		(0.67–1.40)
rs1263171	APOA5-APOA4/ Intergenic	**A**/G	0.5	0.43	7.03	0.008	1.29	0.23^#^	1.16
							(1.07–1.57)		(0.91–1.49)
rs7396835	APOA5-APOA4/ Intergenic	T/C	0.39	0.44	4.03	0.045	0.81	0.03	0.81
							(0.67–0.99)		(0.67–0.99)
rs633867	APOA5-APOA4/ Intergenic	**T**/C	0.07	0.05	3.1	0.078	1.4	0.009	1.96
							(0.96–2.05)		(1.17–3.28)
rs17440396*	BUD13/Intronic	A/G	0.03	0.21	114.9	8.18x10^-27^	0.13	4.42x10^-8^	0.22
							(0.08–0.19)		(0.13–0.38)
rs664059	BUD13/Intronic	**T**/C	0.37	0.3	8.01	0.005	1.34	0.01	1.38
							(1.09–1.64)		(1.06–1.80)
rs10488699	BUD13/Intronic	**A**/G	0.24	0.2	4.19	0.041	1.28	0.54^#^	0.9
							(1.01–1.62)		(0.65–1.25)
rs2187126	BUD13/Intronic	G/A	0.1	0.13	3.88	0.049	0.74	0.50^#^	0.86
							(0.54–0.99)		(0.57–1.31)
rs6589566*	ZPR1/Intronic	**G**/A	0.39	0.25	34.36	4.58x10^-09^	1.87	0.012	1.4
							(1.51–2.32)		(1.07–1.83)
rs2075294	ZPR1/Intronic	**T**/G	0.08	0.05	5.15	0.023	1.56	0.019	1.82
							(1.06–2.31)		(1.10–3.03)

*SNPs significant after multiple corrections

^#^p-value not significant

Allele in bold indicates risk increasing.

Since the 11q23.3 chromosomal region plays a vital role in regulating lipid traits, we analyzed the association of these SNPs adjusting for covariates age, sex and lipid traits, viz. total cholesterol (TC), LDL cholesterol, HDL cholesterol, triglycerides and VLDLs. The results suggest that only 6 out of the 12 SNPs show significant association after adjusting for covariates, suggesting independent effects; while rs664059, rs6589566 and rs2075294 remained significantly associated with increased risk for CAD, rs2849165, rs7396835 and rs17440396 showed decreased risk for CAD (Table 2). In addition to these an *APOA5*-*APOA4* intergenic SNP rs633867-T, which was not associated with CAD earlier, turned out to be significant with increased risk after adjusting for covariates (OR = 1.96(1.17–3.28); p value = 0.009). We further explored possible association of these 12 CAD related variants with the quantitative lipid traits viz., TC, LDLC, HDLC, TG and VLDL, as the main phenotypes. A significant association of rs17440396, rs2187126, rs633389 and rs1262163 is apparent with TC and LDLC implying their risk for dyslipidemia in contrast to rs6589566 that showed negative regression suggesting decreasing risk for dyslipidemia (S3 Table). This trend is consistent with the genotypic distribution of mean levels of LDLC and TC (S1 Fig). However, assuming that the dyslipidemia is the primary trigger for CAD, these trends are somewhat contradictory to the pattern of association of these SNPs with CAD. This may imply that the variants at 11q23.3 chromosomal region exhibit distinct effects in manifesting CAD and/or dyslipidemia.

### Genotypic association with CAD

In order to understand the genotype-phenotype relationship, we performed logistic regression of CAD on the genotypes under different genetic models and the results are provided in Table 3. The best genetic model for the genotypic effect was chosen based on the lowest AIC (akaike information criteria) value. We observed three variants that belong to *APOA5*-*APOA4* intergenic region, rs633389 (OR = 0.49; P value = 1.72x10^-6^), rs1263163 (OR = 0.48; P value = 6.8x10^-8^) and rs2849165 (OR = = 0.47; P value = 1.6x10^-12^) to be significantly associated with CAD under log additive model. While the intronic variant rs17440396 of *BUD13* showed highly significant protective effect under dominant model (AG-AA vs GG; OR = 0.08, P value = 1.4x10^-37^), rs2187126 displayed similar association (AG vs AA-GG; OR = 0.63, P value = 0.007) under over-dominant model. On the contrary, rs6589566 that belongs to *ZPR1* intronic region is found to increase risk towards CAD (AG-GG vs AA; OR = 2.38, P value = 6.04x10^-10^), under dominant model. This analysis suggests possible protective role of *BUD13* regulatory gene against CAD in our population while the other regulatory gene, *ZPR1*, confers susceptibility to CAD. Except for the variant rs2187126, the patterns of association remained similar after adjusting for the covariates (Table 3).

**Table 3:** Genotypic association of variants at 11q23.3 chromosomal region with the CAD along withchi-square values and odds ratios from logistic regression, before and after adjusting for covariates age, sex and lipid traits.

Gene	SNP	Model*	Genotype	Frequency		Unadjusted		Adjusted for age, sex and lipid traits	
				Cases	Control	OR (95% CI)	P-value	OR (95% CI)	P-value
BUD13	rs17440396	Dominant	AG-AA	0.05	0.4	0.0842	1.4x10-^37^	0.11	3.12x10^-20^
			GG	0.95	0.6	(0.05–0.13)		(0.06–0.19)	
BUD13	rs2187126	Overdominant	AG	0.16	0.24	0.63	0.007	0.85	0.44^#^
			AA-GG	0.84	0.76	(0.60–1.06)		(0.56–1.28)	
Intergenic APOA5-APOA4	rs1263163	Log-additive	—	—	—	0.48	6.8x10^-8^	0.67	0.0149
						(0.36–0.63)		(0.48–0.92)	
Intergenic APOA5-APOA4	rs2849165	Log-additive	—	—	—	0.47	1.6x10^-12^	0.46	1.209x10^-9^
						(0.38–0.59)		(0.35–0.59)	
Intergenic APOA5-APOA4	rs633389	Log-additive	—	—	—	0.49	1.72x10^-6^	0.66	0.0196
						(0.36–0.67)		(0.46–0.94)	
ZPR1	rs6589566	Dominant	AG-GG	0.62	0.41	2.38	6.04x10^-10^	2.5	9.71x10^-8^
			AA	0.38	0.59	(1.78–3.15)		(1.75–3.44)	

^#^p-value not significant

*Best genotypic model represented by the variant genotype selected as per lowest AIC value

### Linkage Disequilibrium and Haplotype analysis

**Fig 1:**
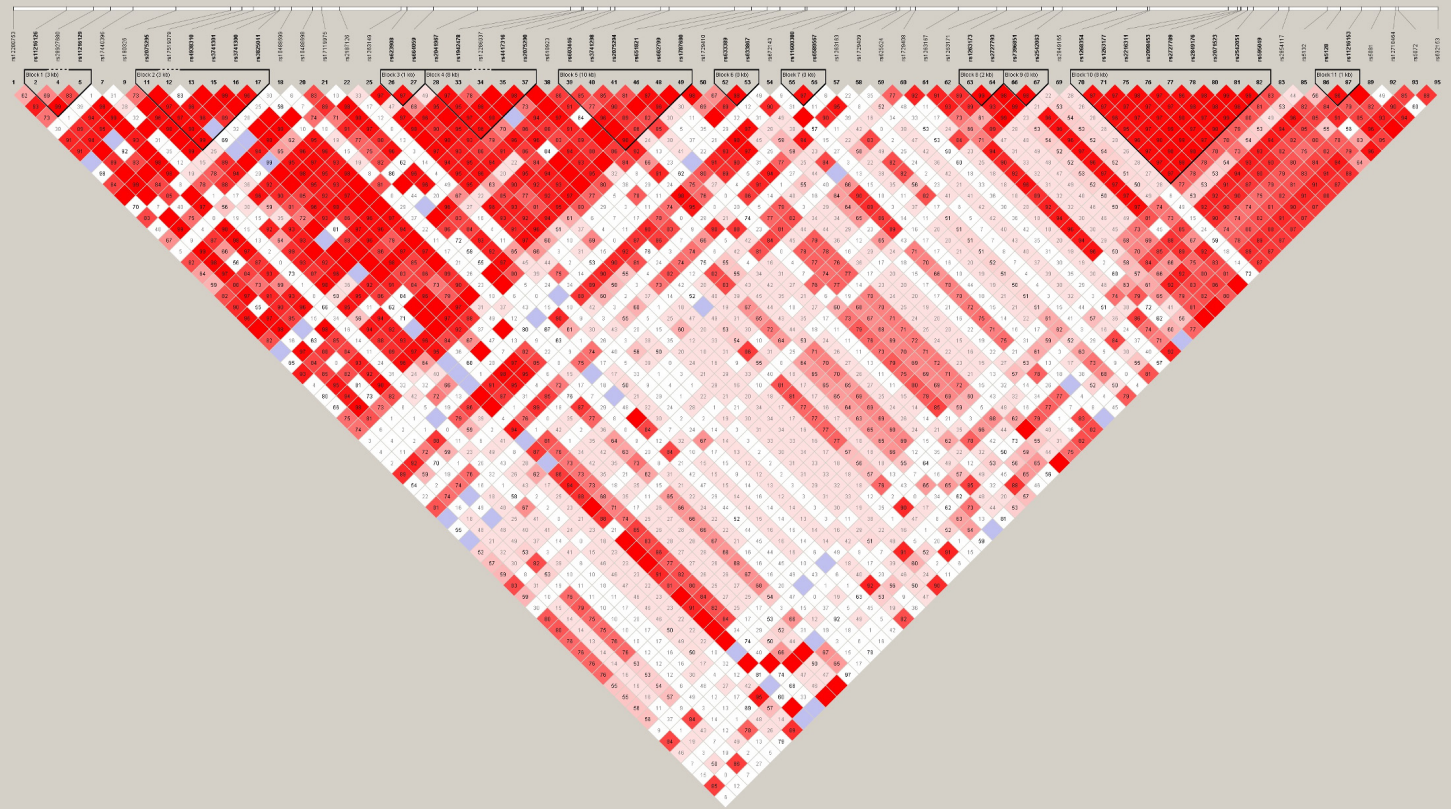
Linkage Disequilibrium plot of SNPs at 11q23.3 chromosomal region encompassing BUD13-ZPR1, APOA1-C3-A4-A5 genes. Pair wise LD among 63 SNPs is indicated by diamonds shaded in white–pink–red, the strength of LD reflected in the intensity of colour, from D’ = 0 in white to D’ = 1 in red. The 11 blocks represent the presence of strong LD.

Pair wise LD analysis of all the SNPs with their D’ values (Fig 1) revealed 55 SNP pairs with r^2^> 0.8, 63 pairs with r^2^ between 0.5 and 0.8 and another 68 SNP pairs with r^2^ < 0.5. Overall, a disrupted LD pattern is seen in this population with 46 tag SNPs identified at r^2^ ≥ 0.8. Using Gabriel et al [26] haplotype block definition criteria, 11 haplotype blocks were identified in this chromosomal region. Table 4 provides the information on SNPs and genomic region covered in each of the haplotype blocks. Table 5 provides frequencies of the associated haplotypes with the respective blocks and constituent SNPs along with the results of logistic regression analysis.

**Table 4:** Details of haplotype blocks at 11q23.3 chromosomal region with their SNPs.

Haplotype Block No	No of SNPs	Rs ID of SNPs	Nucleotide Start Position	Nucleotide End Position	Region Covered in KB
1	2	rs11216126, rs11216129	116746524	116749540	3.017
2	5	rs2075295, rs4938310, rs3741301, rs3741300, rs3825041	116757685	116760991	3.307
3	2	rs623908, rs664059	116769652	116771421	1.77
4	4	rs2041967, rs1942478, rs4417316, rs2075290	116774433	116782580	8.148
5	6	rs603446, rs3741298, rs2075294, rs651821, rs662799, rs1787680	116783719	116794060	10.342
6	2	rs633389, rs633867	116796621	116796764	0.144
7	2	rs11600380, rs6589567	116799466	116799960	0.495
8	2	rs1263173, rs2727793	116810292	116812658	2.367
9	2	rs7396851, rs2542063	116813448	116814051	0.604
10	9	rs1268354, rs1263177, rs2216311, rs2098453, rs2727789, rs2849176, rs2071523, rs2542051, rs595049	116819862	116828729	8.868
11	2	rs5128, rs11216153	116832924	116834384	1.461

Note: Nucleotide start and end positions are with reference to NCBI build GRCh38

**Table 5:** Haplotype association of variants at 11q23.3 chromosomal region with CAD, chi-square values and odds ratios from logistic regression analysis before and after adjusting for age and sex.

Haplotype Block No	SNPs in the haplotype block	Associated Haplotype	Frequency		Χ^2^	Unadjusted		Adjusted	
			Cases	Controls		Odds Ratio	P value	Odds Ratio	P value
3	rs623908, rs664059	A**T**	0.36	0.29	7.26	1.28	0.012	1.19	0.0965*
4	rs2041967, rs1942478, rs4417316, rs2075290	GTC**T**	0.11	0.089	4.07	1.36	0.0492	1.31	0.101*
5	rs603446, rs3741298, rs2075294, rs651821, rs662799, rs1787680	CG**T**TAT	0.073	0.048	4.27	1.57	0.0355	1.59	0.0413
6	rs633389,rs633867	TC	0.005	0.1	70.9	0.041	6.97x10-^10^	0.051	1.25x10-^08^
		CC	0.91	0.84	22	1.96	1.50 x10^-5^	1.69	0.00113
		T**T**	0.077	0.05	2.84	1.36	0.106*	1.49	0.0471

*p- not significant; Alleles in bold indicates risk increasing.

We found haplotypes AT (OR = 1.28; p value = 0.01) and CGTTAT (OR = 1.57; P value = 0.03) carrying risk alleles of the SNPs, rs664059 and rs2075294,from the H3 and H5 haplotype blocks, respectively, as associated with increased risk for CAD. The TC haplotype with SNPs rs633389 and rs633867 that belong to H6 block is found to show decreased risk for CAD (OR = 0.04; P value = 6.97x10^-10^). These patterns of haplotypic association are similar after adjusting for age and sex as covariates. However, after adjusting for age and sex we observed TT haplotype of H6 block to be associated with increased risk for CAD (OR = 1.49; P value = 0.047). This is similar to the allelic association finding from logistic regression analysis, where SNP rs633867 exhibited significantly increased risk for CAD after adjusting for covariates. On the other hand, GTCT haplotype of block H4, which was significantly associated (OR = 1.36; P value 0.049) with increased risk for the disease turns out to be non-significant (P value = 0.1) after adjusting for the covariates. However, no SNP that belongs to this block is found associated at allelic or genotypic level with the disease.

### SNP-SNP interactions

Given that the SNPs under study are within 200kb of 11q23.3 chromosomal region, we attempted to understand the interaction effects among these SNPs on the CAD by standard parametric approach implemented through pair wise logistic regression analysis of the associated 12 SNPs using PLINK. From the pairs of SNPs that showed significant interactions along with the associated P values and odds ratios (Table 6), the following three categories of interactions can be inferred:

1. Interactions between intronic SNPs of *BUD13* regulatory gene i.e., rs17440396, rs10488699, rs2187126 with intergenic SNPs rs633389, rs1263163, rs1263171 of *APOA5*-*APOA4* genes.
2. Intronic SNP, rs6589566 of *ZPR1* gene and *APOA5*-*APOA4* Intergenic SNP rs1263163.
3. Interactions within *APOA5*-*APOA4* intergenic region involving SNP pairs rs1263163-rs123171 and 1263163-rs2849165

**Table 6:** Significant SNP-SNP interaction effects on CAD obtained through pair wise logistic regression.

SNP Pair		Involved Genes	Odds Ratio	P Value
rs17440396	rs633389	IntronicBUD13-IntergenicAPOA5-A4	0.06027	2.33x10^-05^
rs10488699	rs633389	IntronicBUD13-IntergenicAPOA5-A4	0.1559	8.57E x10^-09^
rs10488699	rs1263163	IntronicBUD13-IntergenicAPOA5-A4	0.08845	1.39 x10^-14^
rs2187126	rs633389	IntronicBUD13-IntergenicAPOA5-A4	0.06231	2.05x x10^-11^
rs2187126	rs1263163	IntronicBUD13-IntergenicAPOA5-A4	0.04653	7.21 x10^-14^
rs2187126	rs1263171	IntronicBUD13-IntergenicAPOA5-A4	2.681	1.35 x10^-05^
rs6589566	rs1263163	Intronic ZPR1-Intergenic APOA5-A4	3.548	2.91 x10^-07^
rs1263163	rs1263171	Within APOA5-APOA4	0.4635	3.41 x10^-05^
rs1263163	rs2849165	Within APOA5-APOA4	0.07547	3.90 x10^-14^

Among the identified SNP-SNP interactions, rs2187126-rs1263171 (OR = 2.681, P Value = 1.35 x10^-05^) and rs6589566-rs1263163 (OR = 3.548, P value = 2.91 x10^-07^) that belong to categories one and two above show increased risk for CAD. Both the regulatory genes *BUD13* and *ZPR1* are involved in disease manifestation through their significant interactions with *APOA4*-*APOA5* intergenic region.

### Generalized Multifactor Dimensionality Reduction (GMDR) approach for Interaction analysis:

GMDR is an agnostic and nonparametric approach to determine the gene-gene interactions. It is widely used because of its power to detect interactions in small sample sizes while permitting adjustment for covariates. Here we carried out an exhaustive search over all possible combinations of interactions to an order of six attributes with 10 fold cross validation. However, significant interactions were observed only up to five loci combination, with interaction models above the threshold testing balance accuracy of 0.55 with significant test p-values and high cross validation consistency are selected as the best models. With their consistent occurrence in all interaction models, rs17440396 and rs2849165 emerge as the most prominent variants in the interaction analysis. This implies the presence of significant interaction between the intronic region of *BUD13* gene and intergenic region of *APOA4*-*APOA5* genes, which persists even after adjusting for age and sex (Table 7). Additionally, interaction of rs6589566 of *ZPR1* regulatory gene appeared in 5 loci model. The results of parametric and non-parametric interaction analysis are concurrent in suggesting that the interaction between intergenic variants of *APOA5*-*APOA4* genes and intronic variants of *BUD13*, *ZPR1* regulatory genes plays a major role in the manifestation of CAD in this population.

**Table 7:** Summary results of best SNP-SNP interaction model using GMDR.

Model	Unadjusted				Adjusted for covariates age and sex			
	Balance Accuracy		p-value	CVC	Balance Accuracy		p-value	CVC
	Training	Testing			Training	Testing		
rs17440396	0.6861	0.6855	0.001	10/10	0.6819	0.6808	0.001	10/10
rs2849165, rs17440396,	0.7315	0.7283	0.001	10/10	0.7296	0.7250	0.001	10/10
rs2849165, rs17440396, rs1263163	0.7828	0.7732	0.001	10/10	0.7812	0.7697	0.001	10/10
rs10488699, rs2849165, rs2187126, rs17440396	0.7990	0.7759	0.001	8/10	0.7979	0.7796	0.001	9/10
rs10488699, rs2849165, rs7396835, rs17440396, rs6589566	0.8208	0.8003	0.001	10/10	0.8196	0.8196	0.001	10/10

CVC: Cross Validation Consistency

### Results of cumulative risk score analysis for CAD associated variants

**Fig 2:**
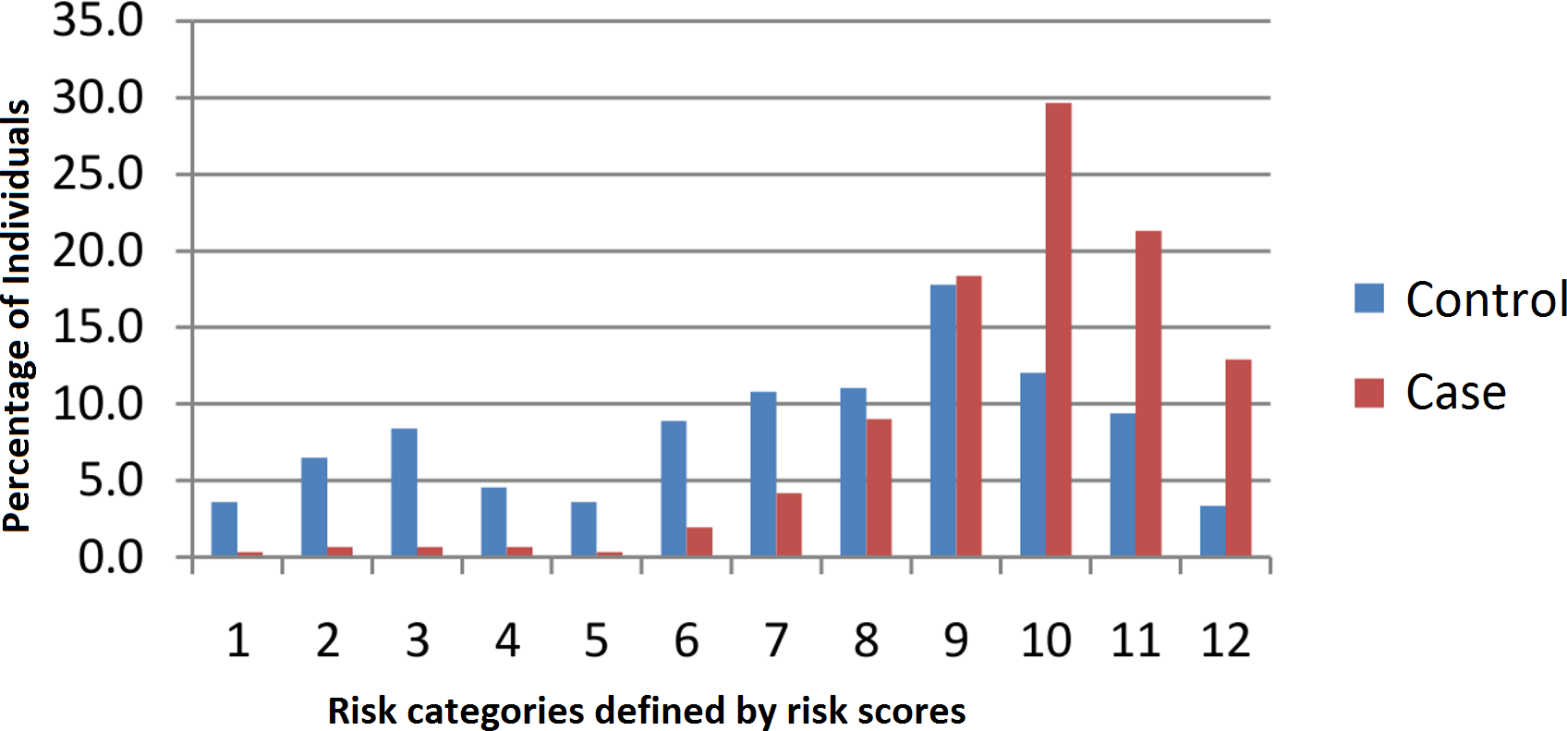
Relative proportion of cases and controls in the 12 risk categories

**Fig 3:**
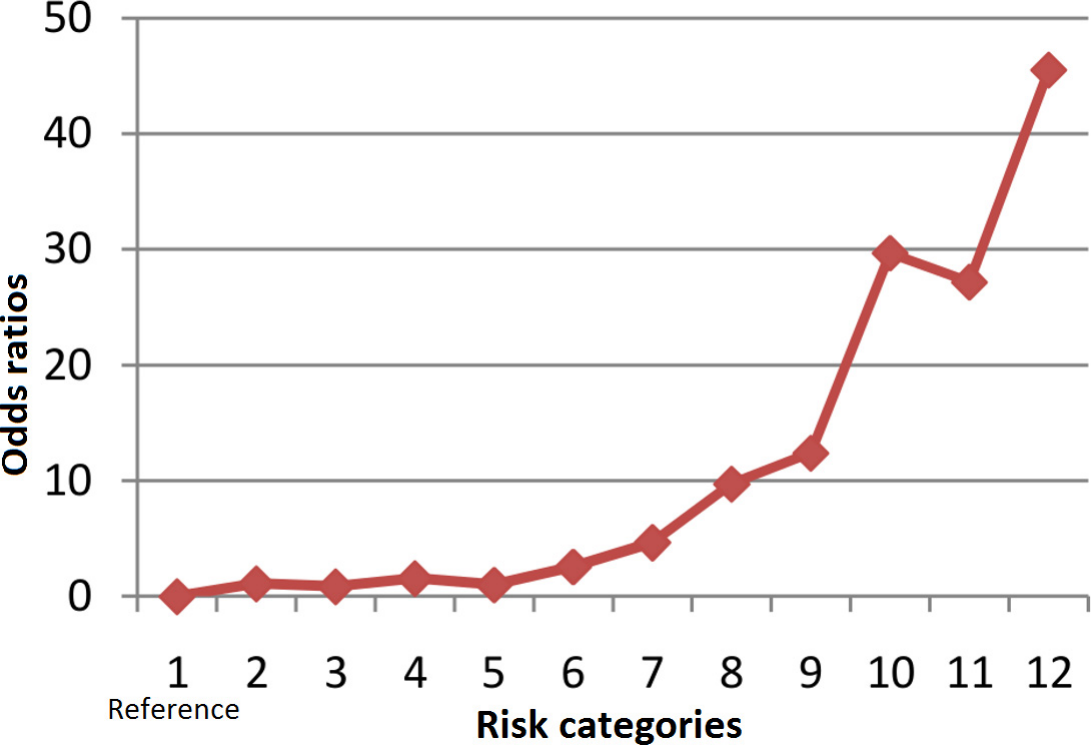
Plot of odds ratios according to risk categories

**Fig 4:**
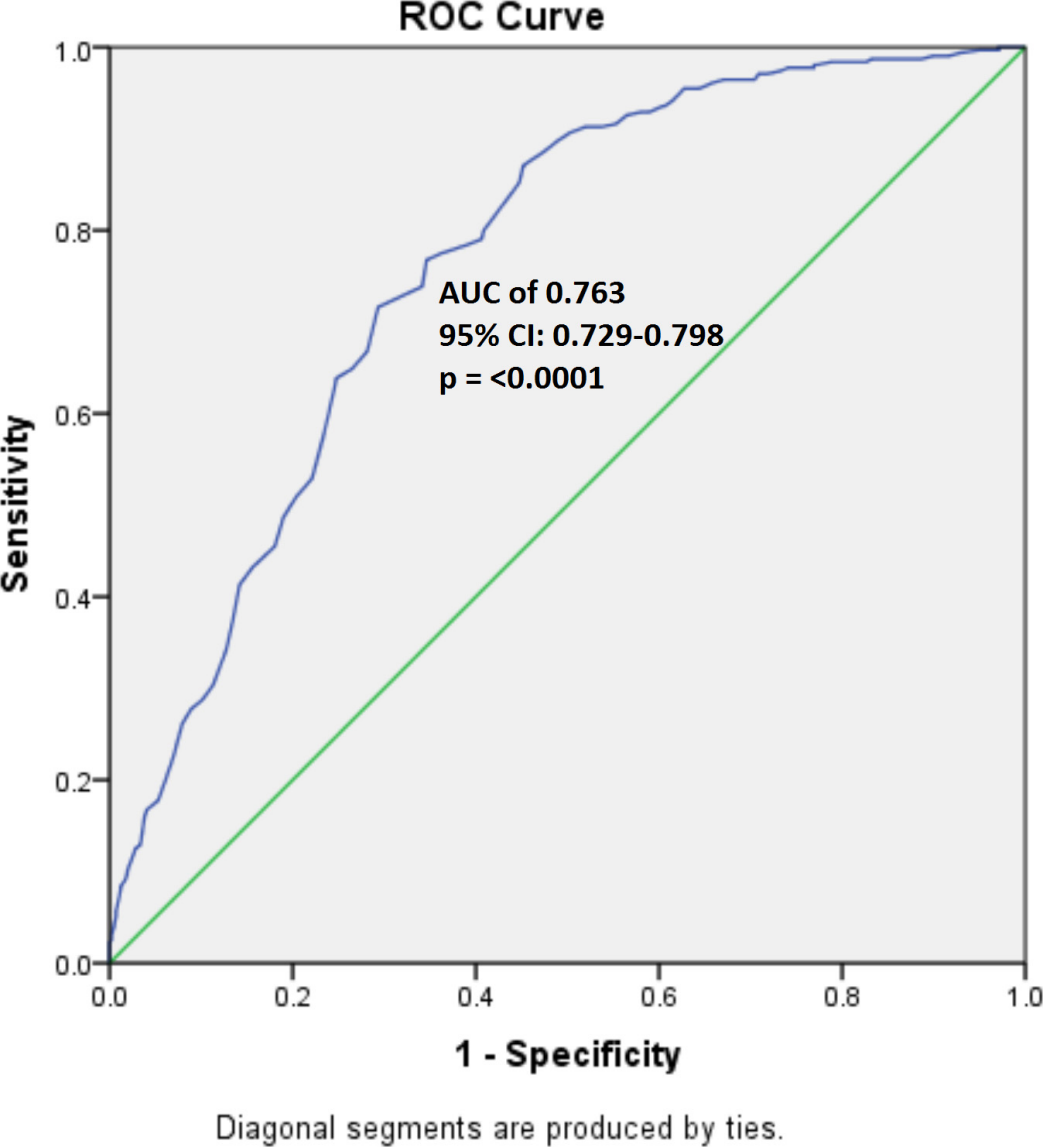
Receiver Operating Curve indicating the area under curve (AUC) and the discriminative power of risk scores

In order to determine the combined risk effect of the 12 significant SNPs, we computed the weighted mean proportion of the risk alleles at these 12 SNPs by taking 2 for two risk alleles, 1 for one risk allele and 0 for no risk alleles with weights as relative log odds ratios of different SNPs. The cumulative risk allele score for each individual is obtained by multiplying with 12 (no of SNPs in analysis). The individuals with the risk scores ranging from 2.7 to 20.9 were grouped into 12 risk categories as shown in Table 8. Given the very low frequency of Individuals within the risk score range of 2–9 and 19–21, these were merged into the categories 1 and 12, respectively. Fig 2 shows the distribution of CAD cases and controls according to the risk category. A clear trend of increased percentage of cases with increasing risk score is observed. With reference to risk category 1, we computed odds ratios for each of the remaining 11 risk categories. An increasing trend of OR values with increasing number of risk alleles is apparent from the plot (Fig 3). However, the odds ratios suggest significant association of CAD only with risk categories ranging from 7–12. Further, to understand the discriminative power of the risk scores, we constructed the ROC (receiver operating curve) plot (Fig 4) for the risk scores and CAD status, which yielded area under curve (AUC) as 0.763 (95% CI: 0.729–0.798, p = <0.0001). Given these highly significant results, the observed AUC probably indicates that this study has substantial power to confer these genetic variants as predictors of risk for CAD.

**Table 8:** Percentage of CAD cases and controls with cumulative risk scores for CAD associated variants and results of logistic regression analysis of risk categories on CAD.

RISK CATEGORY	Risk Score	Percentage of Individuals in Controls	Percentage of Individuals in Cases	Odds ratio	Z-score	P value
1	2–9	3.6	0.3	Reference	-	
2	9–10	6.5	0.6	1.1 (0.26–4.69)	0.139	0.889
3	10–11	8.4	0.6	0.86(0.20–3.61)	0.21	0.833
4	11–12	4.6	0.6	1.56 (0.36–6.69)	0.604	0.54
5	12–13	3.6	0.3	1 (0.18–5.28)	0	1
6	13–14	8.9	1.9	2.56(0.71–9.1)	1.44	0.149
7	14–15	10.8	4.2	4.6 (1.3–15.9)	2.454	0.01*
8	15–16	11.1	9.0	9.7 (2.9–32.6)	3.68	0.0002*
9	16–17	17.8	18.4	12.4 (3.75–41.0)	4.12	<0.0001*
10	17–18	12.0	29.7	29.7 (8.97–98.2)	5.554	<0.0001*
11	18–19	9.4	21.3	27.19 (8.16–90.5)	5.383	<0.0001*
12	19–21	3.4	12.9	45.5 (13.2–156.8)	6.05	<0.0001*

*significant p-value

## Discussion

The apo genes clustered at 11q23.3 chromosomal region codes for plasma apolipoproteins *APOA1*, *APOC3*, *APOA4* and *APOA5*. *APOA1*, as a major component of HDL and cofactor for lecthin cholesterol acyl transferase (LCAT) enzyme, is an established biochemical marker of CAD. *APOC3* is a component of VLDL and inhibits lipase enzyme. *APOA4* was found to activate LCAT enzyme. *APOA5*, being a component of VLDL, HDL and chylomicrons, was observed to influence plasma triglyceride levels. With these functional implications in lipoprotein metabolism, several polymorphisms at 11q23.3 were associated with elevated plasma lipoprotein and cholesterol levels and in turn also with CAD. So far, Indian studies pertaining to the above genes could validate only the conventional polymorphisms of *APOA5*-1131T>C (rs662799), −3A>G (rs651821), S19W (rs3135506), G185C (rs2075291) and *APOC3*-SacI SNP (rs5128) [6, 15, 16, 27]. On the other hand, a GWAS conducted on a sample of glycemic and non glycemic subjects, as part of Sikh Diabetes Study (SDS),Sanghera et al [8] observed an intronic variant of *ZPR1* regulatory gene rs12286037 to be associated with triglycerides. This study also validated rs964184, the most consistently replicated *ZPR1*-*BUD13* intergenic SNP across the ethnic groups. Further, exploring the association pattern of 45 SNPs located between the above two SNPs, they found five SNPs- rs7350481, rs180326, rs618923, rs10047459 and rs533556- to be associated with plasma triglycerides [8]. Similarly, Gagandeep et al. [28] observed rs964184 and rs662799 to be associated with triglycerides among the Indian subjects screened as part of the Indian migration study (IMS). However, despite their characteristic atherogenic dyslipidemic feature, the south Indian populations were not hitherto explored for these variants. Our attempt to comprehensively explore the pattern of association of variants at 11q23.3 chromosomal region with CAD, however, yielded a different set of SNPs in the population of Hyderabad. Of the genetic variants that belong to *APOA5*-*APOA4* intergenic region, rs2849165-A, rs1263163-A and rs633389-T show a profound risk reducing effect on CAD in our population, which is perhaps a pioneering observation. That the association of rs633867-Twas significant only when adjusted for lipid traits may imply its independent risk conferring nature to CAD, which was probably masked by the confounding effects of lipid traits. This variant along with another tag SNP rs633389-T forms a significant risk conferring haplotype as well. There is also an interaction among the variants of *APOA5-A4* intergenic region on the one hand and these variants with the variants of *BUD13* and *ZPR1* regulatory genes on the other. Of the four *BUD13* SNPs, we observed rs17440396-A to be significant even after adjusting for lipid traits and regulates several associated SNPs through its interaction. Further, a bivariate genome wide approach among Europeans with metabolic syndrome has identified three SNPs (rs11825181, rs11820589, and rs10790162) of this gene to be associated with trait combinations TG-BP, TG-GLUC, and TG-HDLC, MetS and WC-TG, respectively [29]. Subsequently, one of these variants (rs10790162) was also found associated with TC among the Chinese [30]. However, none of these were found to be associated with CAD in our population. With its evolutionarily conserved function of forming pre m-RNA Retention and Splicing (RES) complex [31], *BUD13* appears to be one of the key regulating genes of 11q23.3 chromosomal region. Our results of both GMDR and SNP-SNP pair wise interaction analysis are congruent with this in showing significant interaction of rs17440396 *BUD13* intronic variant with that of intergenic *APOA5*-*APOA4* and intronic variantsof*ZPR1* genes. Conversely, an intronic variant rs6589566-G of *ZPR1*gene is observed to exhibit high risk for CAD in the population of AP. This variant is found to interact independently with *APOA5*-*APOA4* intergenic region as well as in combination with variants of *BUD13* gene. It is found to be associated with triglycerides and LDL cholesterol among Europeans [32, 33] and with triglycerides, in conjunction with a downstream 500bp variant rs964184 (D’ = 1, r2 = 1), among the Han Chinese. A Meta analysis of 14 GWAS studies on Europeans identified SNP rs964184 of *ZPR1* gene to be significantly associated with HDL cholesterol, coronary artery disease [22] and other lipid traits among Asian Indians and Chinese [8,30]. However, we did not find any association of this SNP with either CAD or any of the lipid traits in the present study. Another variant of this gene, rs2075294-T, is shown to be susceptible to CAD in our population at allelic level albeit not at the genotype level. It was observed that the deficiency of *ZPR1* protein causes defects in transcription and cell cycle progression [34] due to improper localization of survival motor neuron (SMN1) protein, gems and cajal bodies. Therefore, it is imperative to understand the precise role of the variants at *ZPR1* gene in regulating the apolipoprotein levels. In particular, the risk conferring nature of rs6589566 of this gene towards CAD both at the allelic and genotypic levels as well as by its interaction with rs1263163 of *APOA5*-*APOA4* intergenic region in our population probably warrants functional validation. Overall, we found that the variants associated with CAD in the present study are unique. By far the most significant result could be the high discriminative power of the risk scores as illustrated by AUC (0.763 (95% CI: 0.729–0.798, p = <0.0001), suggesting that our study has substantial power to confer these genetic variants as predictors of risk for CAD.

### Distinct patterns of association of SNPs at 11q23.3 region with CAD and Dyslipidemia

Except for the association of *APOA1* gene polymorphism rs5072 (756 C>T) at the allelic level, the earlier reported conventional polymorphisms of this chromosomal region are not associated in the present study. Although this is consistent with the findings of a study from Western India [16], the urban study of Chennai on the contrary suggests association of two conventional polymorphisms with CAD [35]. However, the meta analysis on the 3 SNPs (rs5128, rs662799 and rs651821) for which comparative data were available from the above two populations [16, 35] (S4 Table) suggests the overall effect (under the random effect model) to be non-significant for any of the three SNP which implies that these conventional polymorphisms may not have a direct risk conferring effect on CAD in the studied populations. This may suggest that the atherogenic dyslipidemia, with putative role in manifesting CAD in this population, has its specific genetic etiological background. In this context, it may be plausible to surmise if the regulatory genes of 11q23.3 chromosomal region that are found associated with CAD contain a pool of both upregulating and downregulating genetic variants for the lipid traits. Our quantitative trait analysis is concurrent with this conjecture, albeit the risk reducing variants of CAD are risk conferring towards dyslipidemia by their effect in elevating LDLC and TC. However, this might be a result of confounding effects of lower average levels of these lipid traits observed in the case cohort. In order to determine true association of the above variants with lipid traits we tested for their association with dyslipidemia in the control cohort and found that the results are congruent with the susceptible nature of the CAD protective variants to dyslipidemia (S5 Table). Further, the test of genetic association with CAD cases and nondyslipidemic controls yielded identical results compared to what was obtained with pooled sample of controls that included significant proportion of dyslipidemic subjects. The distinct effects of these variants observed in manifesting CAD and dyslipidemia suggest that there is a direct role of the *APOA4*-*APOA5* intergenic variants of this region in regulating lipid traits. Given the pleiotropic nature of *BUD13* and *ZPR1* regulatory genes, an indirect risk reducing effect of these genes towards CAD could possibly be a result of their action mediated through other metabolic pathways such as inflammation, oxidative stress, thrombosis, blood glucose homeostasis etc. The interactions between the genes might also cause these varying effects of SNPs. For example, rs2187126, which is a protective variant of *BUD13* regulatory gene show profound risk towards CAD through its interaction with *APOA5*-*APOA4* intergenic risk variant rs1263171 (OR = 2.68; P value = 1.35 x10-05). Also interaction between rs6589566 and rs1263163 is observed to confer increased risk towards CAD (OR = 3.54; P value = 2.91 x10-07). Functional analysis of these variants would probably provide better insights into the precise roles of these genes in the 11q23.3 chromosomal region.

## Conclusion

We found unique variants of 11q23.3 apo gene cluster region as associated with coronary artery disease in this population, which could be because of the differential genetic predisposition of Indians to complex genetic disorders, as compared to other ethnic groups. This was amply demonstrated in a few earlier studies with reference to cardiomyopathies [36], recurrent miscarriages [37], PCOS [38] and T2DM [39, 40, 41]. Most of the variants we observed to be associated with CAD in the present study belong to *APOA5*-*APOA4* intergenic region and intronic regions of *BUD13* and *ZPR1* genes. Although these genes were reported earlier to have remarkable associations with lipid traits, we validated their significant effect in manifesting CAD. The most significant finding of our study is the high discriminative power of the risk scores based on the 12 significant SNPs, as illustrated by AUC (0.763 (95% CI: 0.729–0.798, p = <0.0001). This may suggest that these genetic variants possess substantial power to be the predictors of risk for CAD.

This study also demonstrated significant interaction between regulatory genes of 11q23.3 region and intergenic *APOA5*-*APOA4* protein coding genes to show distinct effects in manifesting CAD and dyslipidemia. Future studies on genetic association of CAD should include SNPs related to other metabolisms along with the above regulatory genes to get better insights into the mechanisms leading to the distinct effects of the genes observed in the present study. We are in the process of generating high throughput genotype data representing important pathways responsible for atherosclerosis as well as the other genes of lipoprotein metabolism. It may be pertinent to extrapolate this approach of focusing on disease specific domains of genome to other complex diseases which may help identifying important population specific and disease specific genetic markers. More aptly, a GWAS covering the broad ethnic elements in the Indian population with sizeable sample would provide genetic susceptibility profile for CAD at large.

## Supporting Information

S1 Fig Genotype wise mean levels of quantitative lipid traits for significant SNPs of 11q23.3 chromosomal region associated with them. (TIF)

S1 Table Localized gene, nucleotide position and biological relevance of SNPs at 11q23.3 chromosomal region. (DOCX)

S2 Table Allelic and genotype data for CAD cases and controls for all the SNPs under study. (XLSX)

S3 Table Results of linear regression of CAD related variants at 11q23.3 with quantitative lipid traits in the pooled sample of cases and controls. Footnote: β (Beta) indicates the linear regression coefficient. SE indicates standard.
(DOCX)

S4 Table Meta-analysis of SNPs at 11q23.3 chromosomal region analyzed under random effect model. Footnote: *Conventional ID of the SNP, MAF Minor Allele Frequency.
(DOCX)

S5 Table Comparative association analysis of CAD cases and controls in three sets of subjects. Footnote: *Set 1 indicates CAD cases vs controls, Set 2 indicates CAD cases vs non dyslipidemic controls, Set 3 indicates dyslipidemic controls vs non dyslipidemic controls. # indicates p value not significant.
(DOCX)
