# Supplementary figures and images for: Distinct Patterns of Association of Variants at 11q23.3 Chromosomal Region with Coronary Artery Disease and Dyslipidemia in the Population of Andhra Pradesh, India
Source: PLoS One. 2016 Jun 3;11(6):e0153720. doi: 10.1371/journal.pone.0153720 (PMC4892567; doi:10.1371/journal.pone.0153720)

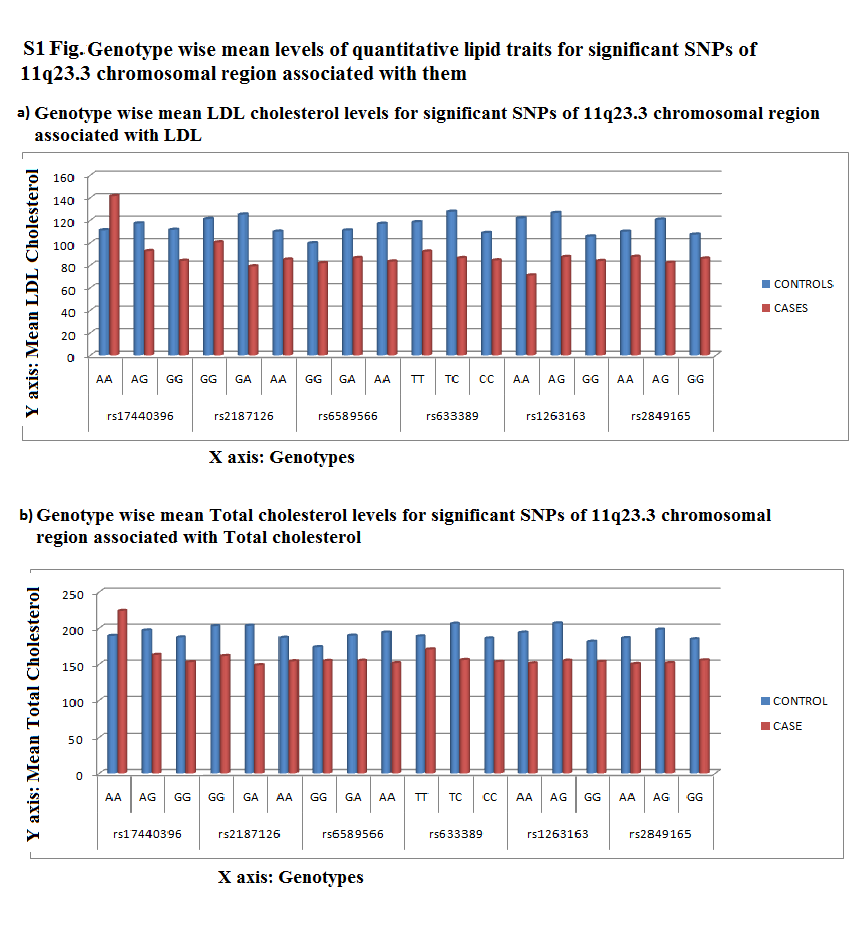

Supplement: S1 Fig — (TIF) [file pone.0153720.s001.tif]
