# Supplementary material for: Distinct Patterns of Association of Variants at 11q23.3 Chromosomal Region with Coronary Artery Disease and Dyslipidemia in the Population of Andhra Pradesh, India
Source: PLoS One. 2016 Jun 3;11(6):e0153720. doi: 10.1371/journal.pone.0153720 (PMC4892567; doi:10.1371/journal.pone.0153720)
Supplement: S1 Table — (DOCX) [file pone.0153720.s002.docx]

**S1 Table. Localized gene, nucleotide position and biological relevance of SNPs at 11q23.3 chromosomal region**

| **SNP Rs ID** | **Gene** | **Biological relevance** | **Nucleotide Position** |
| --- | --- | --- | --- |
| rs12280753 | LOC10192011-BUD13 | Intergenic | 116742944 |
| rs11216126 |  | Intergenic | 116746524 |
| rs11556024 | BUD13 | Utr3 | 116748335 |
| rs28927680 |  | Utr3 | 116748357 |
| rs11216129 |  | Intron | 116749540 |
| rs180338 |  | Intron | 116749977 |
| rs17440396 |  | Intron | 116751054 |
| rs12292921 |  | Intron | 116751247 |
| rs180326 |  | Intron | 116753987 |
| rs180324 |  | Intron | 116754616 |
| rs2075295 |  | Intron | 116757685 |
| rs17519079 |  | Intron | 116758437 |
| rs4938310 |  | Intron | 116759233 |
| rs918143 |  | Intron | 116759884 |
| rs3741301 |  | Intron | 116760675 |
| rs3741300 |  | Intron | 116760974 |
| rs3825041 |  | Intron | 116760991 |
| rs10488699 |  | Intron | 116761784 |
| rs918144 |  | Synonymous | 116763109 |
| rs10488698 |  | Missense | 116763231 |
| rs17119975 |  | Intron | 116763841 |
| rs2187126 |  | Intron | 116765068 |
| rs10790162 |  | Intron | 116768388 |
| rs1268353 |  | Intron | 116768976 |
| rs1263149 |  | Intron | 116769225 |
| rs623908 |  | Intron | 116769652 |
| rs664059 |  | Intron | 116771421 |
| rs2041967 |  | Upstream 2kb | 116774433 |
| rs964184 | ZPR1 | Downstream 500b | 116778201 |
| rs11823543 |  | Downstream 500b | 116778419 |
| rs1043740 |  | Utr3 | 116778795 |
| rs17120029 |  | Intron | 116779402 |
| rs1942478 |  | Intron | 116780747 |
| rs12286037 |  | Intron | 116781491 |
| rs4417316 |  | Intron | 116781585 |
| rs6589566 |  | Intron | 116781707 |
| rs2075290 |  | Intron | 116782580 |
| rs618923 |  | Intron | 116783443 |
| rs603446 |  | Intron | 116783719 |
| rs3741298 |  | Intron | 116786845 |
| rs2075294 |  | Intron | 116787406 |
| rs2266788 | APOA5-ZPR1 | Upstream 2kb, utr3 | 116789970 |
| rs619054 | APOA5 | Utr3 | 116790097 |
| rs2072560 |  | Intron | 116791110 |
| rs3135506 |  | Missense | 116791691 |
| rs651821 |  | Utr5 | 116791863 |
| rs648450 |  | Intron, upstream | 116792069 |
| rs662799 |  | Upstream 2kb | 116792991 |
| rs1787680 |  | Upstream 2kb | 116794060 |
| rs1729410 | APOA5-APOA4 | Intergenic | 116794945 |
| rs7128182 |  | Intergenic | 116795599 |
| rs633389 |  | Intergenic | 116796621 |
| rs633867 |  | Intergenic | 116796764 |
| rs672143 |  | Intergenic | 116799345 |
| rs11600380 |  | Intergenic | 116799466 |
| rs6589567 |  | Intergenic | 116799960 |
| rs1263163 |  | Intergenic | 116802796 |
| rs1729409 |  | Intergenic | 116803052 |
| rs625524 |  | Intergenic | 116803787 |
| rs1729408 |  | Intergenic | 116804102 |
| rs1263167 |  | Intergenic | 116807007 |
| rs1263171 |  | Intergenic | 116809614 |
| rs1263173 |  | Intergenic | 116810292 |
| rs2727793 |  | Intergenic | 116812658 |
| rs7396835 |  | Intergenic | 116813312 |
| rs7396851 |  | Intergenic | 116813448 |
| rs2542063 |  | Intergenic | 116814051 |
| rs2849168 |  | Intergenic | 116814485 |
| rs2849165 |  | Intergenic | 116817762 |
| rs1268354 |  | Intergenic | 116819862 |
| rs1263177 |  | Intergenic | 116819996 |
| rs5110 | APOA4 | Missense | 116820918 |
| rs5095 |  | Intron | 116822447 |
| rs5091 |  | Utr5 | 116823289 |
| rs2216311 | APOA4-APOC3 | Intergenic | 116825327 |
| rs2098453 |  | Intergenic | 116825964 |
| rs2727789 |  | Intergenic | 116826171 |
| rs2849176 |  | Intergenic | 116826205 |
| rs2849174 |  | Intergenic | 116826350 |
| rs2071523 |  | Intergenic | 116826628 |
| rs2542051 |  | Intergenic | 116827022 |
| rs2071521 |  | Intergenic | 116827132 |
| rs595049 | APOC3 | Upstream 2kb | 116828729 |
| rs2854117 |  | Upstream 2kb | 116829426 |
| rs2854116 |  | Upstream 2kb | 116829453 |
| rs5132 |  | Intron | 116832062 |
| rs5128 |  | Utr3 | 116832924 |
| rs11216153 | APOC3-APOA1 | Intergenic | 116834384 |
| rs12718462 |  | Intergenic | 116835003 |
| rs5081 | APOA1 | Downstream 500b | 116835630 |
| rs4882 |  | Missense | 116836220 |
| rs5077 |  | Missense | 116836236 |
| rs12718464 |  | Intron | 116836685 |
| rs5072 |  | Intron | 116836867 |
| rs2070665 |  | Intron | 116836968 |
| rs632153 |  | Upstream 2kb | 116839523 |

Utr - Untranslated region
