# Supplementary material for: Distinct Patterns of Association of Variants at 11q23.3 Chromosomal Region with Coronary Artery Disease and Dyslipidemia in the Population of Andhra Pradesh, India
Source: PLoS One. 2016 Jun 3;11(6):e0153720. doi: 10.1371/journal.pone.0153720 (PMC4892567; doi:10.1371/journal.pone.0153720)
Supplement: S3 Table — Footnote: β (Beta) indicates the linear regression coefficient. SE indicates standard. (DOCX) [file pone.0153720.s004.docx]

**S3 Table. Results of linear regression of CAD related variants at 11q23.3 with quantitative lipid traits in the pooled sample of cases and controls**

| Gene | Associated SNP(Minor Allele) | LDLC | | | TCHOL | | |
| --- | --- | --- | --- | --- | --- | --- | --- |
|  |  | **β** | **SE** | **P value** | **β** | **SE** | **P value** |
| BUD13 | rs17440396(A) | 16.3  (11..5-21.1) | 2.469 | 7.23x10^-11^ | 22.26  (16.6-27.9) | 2.896 | 4.43x10^-14^ |
|  | rs2187126(G) | 9.509  (4.5-14.5) | 2.557 | 0.0002 | 10.62  (4.7-16.5) | 3.026 | 0.0004 |
| APOA5-APOA4 | rs1263163(A) | 14.49  (10.02-18.96) | 2.282 | 3.59x10^-10^ | 16.81  (11.5-22.1) | 2.703 | 7.97x10^-10^ |
|  | rs633389(T) | 14.17  (9.5-18.9) | 2.401 | 5.26x10^-09^ | 15.78  (1.2-21.3) | 2.848 | 4.05x10^-08^ |
|  | rs2849165(A) | 6.673  (3.1-10.24) | 1.821 | 0.0002 | 7.232  (2.9-11.5) | 2.161 | 0.0008 |
| ZPR1 | rs6589566(G) | -7.911  (-11.2 - -4.6) | 1.693 | 3.51x10^-06^ | -8.416  (-12.35 - -4.4) | 2.005 | 0.00003 |

β (Beta) indicates the linear regression coefficient. SE indicates standard error
