# Supplementary material for: Distinct Patterns of Association of Variants at 11q23.3 Chromosomal Region with Coronary Artery Disease and Dyslipidemia in the Population of Andhra Pradesh, India
Source: PLoS One. 2016 Jun 3;11(6):e0153720. doi: 10.1371/journal.pone.0153720 (PMC4892567; doi:10.1371/journal.pone.0153720)
Supplement: S4 Table — Footnote: *Conventional ID of the SNP, MAF Minor Allele Frequency. (DOCX) [file pone.0153720.s005.docx]

**S4 Table. Meta-analysis of SNPs at 11q23.3 chromosomal region analyzed under random effect model**

| **SNP** | **Gene** | **Study Population** | **No of Cases** | **No of Controls** | **MAF** | | **Allelic OR (95% CI)** | **P Value** |
| --- | --- | --- | --- | --- | --- | --- | --- | --- |
|  |  |  |  |  | **CONTROL** | **CASE** |  |  |
| rs5128  (3238C>G)* | APOC3 | Southern India (Chennai) | 416 | 416 | 0.26 | 0.31 | 1.29  (1.04-1.59) | 0.02 |
|  |  | Western India (Mumbai) | 90 | 150 | 0.27 | 0.34 | 2.34  (0.98-5.5) | 0.067 |
|  |  | Andhra Pradesh (Hyderabad) | 386 | 462 | 0.37 | 0.33 | 0.8  (0.69-1.03) | 0.09 |
|  |  | **Overall Effect**  **Heterogeneity: Tau^2^ = 0.02, Chi^2^ = 10.47, P = 0.0005** | | | | | **1.07**  **(0.88-1.30)** | **0.51** |
| rs662799  (1131T>C)* | APOA5 | Southern India (Chennai) | 416 | 416 | 0.23 | 0.32 | 1.5  (1.21-1.86) | 0.01 |
|  |  | Western India (Mumbai) | 90 | 150 | 0.2 | 0.18 | 0.77  (0.44-1.35) | 0.401 |
|  |  | Andhra Pradesh (Hyderabad) | 386 | 462 | 0.19 | 0.18 | 0.9  (0.73-1.18) | 0.56 |
|  |  | **Overall Effect**  **Heterogeneity: Tau^2^ = 0.02, Chi^2^ = 6.34, P = 0.04** | | | | | **1.03(0.86-1.23)** | **0.79** |
| rs651821  (3 A>G)* | APOA5 | Western India (Mumbai) | 90 | 150 | 0.2 | 0.18 | 0.77  (0.44-1.35) | 0.401 |
|  |  | Andhra Pradesh (Hyderabad) | 386 | 462 | 0.19 | 0.18 | 0.9  (0.73-1.19) | 0.6 |
|  |  | **Overall Effect**  **Heterogeneity: Tau^2^ = 0.00, Chi^2^ = 0.18, P = 0.67** | | | | | **0.94**  **(0.81-1.08)** | **0.37** |

* Conventional ID of the SNP, MAF Minor Allele Frequency
