# Supplementary material for: Distinct Patterns of Association of Variants at 11q23.3 Chromosomal Region with Coronary Artery Disease and Dyslipidemia in the Population of Andhra Pradesh, India
Source: PLoS One. 2016 Jun 3;11(6):e0153720. doi: 10.1371/journal.pone.0153720 (PMC4892567; doi:10.1371/journal.pone.0153720)
Supplement: S5 Table — Footnote: *Set 1 indicates CAD cases vs controls, Set 2 indicates CAD cases vs non dyslipidemic controls, Set 3 indicates dyslipidemic controls vs non dyslipidemic controls. # indicates p value not significant. (DOCX) [file pone.0153720.s006.docx]

**S5 Table. Comparative association analysis of CAD cases and controls in three sets of subjects**

| ASSOCIATION ANALYSIS | Set 1* | | Set 2* | | Set 3* | |
| --- | --- | --- | --- | --- | --- | --- |
| Sample size | **386/462** | | **386/270** | | **192/270** | |
| SNP | **OR(95% CI)** | **P value** | **OR(95% CI)** | **P value** | **OR(95% CI)** | **P value** |
| rs17440396(A) | 0.13  (0.08 - 0.19) | 8.17x10-27 | 0.14  (0.09 – 0.233 | 1.15x10^-15^ | 1.43  (1.04-1.98) | 0.025 |
| rs2187126(G) | 0.73  (0.54 - 0.99) | 0.048 | 1.07  (0.754-1.53) | 0.68^#^ | 2.21  (1.50-3.25) | 4.07x10^-05^ |
| rs1263163(A) | 0.69  (0.53 – 0.89) | 0.0042 | 1.10  (0.79-1.53) | 0.55^#^ | 2.57  (1.85-3.57) | 7.16x10^-09^ |
| rs633389(T) | 0.47  (0.35 – 0.65) | 2.8x10^-06^ | 0.70  (0.49-1.01) | 0.057^#^ | 2.10  (1.47-3.02) | 3.72x10^-05^ |
| rs2849165(A) | 0.49  (0.39 – 0.61) | 2.48x10^-10^ | 0.54  (0.42-0.69) | 1.32x10^-06^ | 1.15  (0.89 – 1.51) | 0.3^#^ |
| rs6589566(G) | 1.87  (1.51-2.32) | 4.58x10^-09^ | 1.49  (1.18-1.87) | 0.0006 | 0.62  (0.45-0.85) | 0.003 |

*Set 1 indicates CAD cases vs controls, Set 2 indicates CAD cases vs non dyslipidemic controls, Set 3 indicates dyslipidemic controls vs non dyslipidemic controls. # indicates p value not significant.
